# Modelling metastatic colonization of cholangiocarcinoma organoids in decellularized lung and lymph nodes

**DOI:** 10.3389/fonc.2022.1101901

**Published:** 2023-01-18

**Authors:** Gilles S. van Tienderen, Marije E. A. van Beek, Ivo J. Schurink, Oskar Rosmark, Henk P. Roest, Jantine Tieleman, Jeroen Demmers, Iain Muntz, James Conboy, Gunilla Westergren-Thorsson, Gijsje Koenderink, Luc JW van der Laan, Monique M. A. Verstegen

**Affiliations:** ^1^ Department of Surgery, Erasmus MC Transplant Institute, University Medical Center Rotterdam, Rotterdam, Netherlands; ^2^ Lung Biology, Department of Experimental Medical Science, Lund University, Lund, Sweden; ^3^ Proteomics Center, Erasmus University Medical Center, Rotterdam, Netherlands; ^4^ Department of Bionanoscience, Kavli Institute of Nanoscience Delft, Delft University of Technology, Delft, Netherlands

**Keywords:** metastatic colonization, decellularization, Cholangiocarcinoma, tumor organoids, extracellular matrix

## Abstract

Cholangiocarcinoma (CCA) is a type of liver cancer with an aggressive phenotype and dismal outcome in patients. The metastasis of CCA cancer cells to distant organs, commonly lung and lymph nodes, drastically reduces overall survival. However, mechanistic insight how CCA invades these metastatic sites is still lacking. This is partly because currently available models fail to mimic the complexity of tissue-specific environments for metastatic CCA. To create an *in vitro* model in which interactions between epithelial tumor cells and their surrounding extracellular matrix (ECM) can be studied in a metastatic setting, we combined patient-derived CCA organoids (CCAOs) (n=3) with decellularized human lung (n=3) and decellularized human lymph node (n=13). Decellularization resulted in removal of cells while preserving ECM structure and retaining important characteristics of the tissue origin. Proteomic analyses showed a tissue-specific ECM protein signature reflecting tissue functioning aspects. The macro and micro-scale mechanical properties, as determined by rheology and micro-indentation, revealed the local heterogeneity of the ECM. When growing CCAOs in decellularized lung and lymph nodes genes related to metastatic processes, including epithelial-to-mesenchymal transition and cancer stem cell plasticity, were significantly influenced by the ECM in an organ-specific manner. Furthermore, CCAOs exhibit significant differences in migration and proliferation dynamics dependent on the original patient tumor and donor of the target organ. In conclusion, CCA metastatic outgrowth is dictated both by the tumor itself as well as by the ECM of the target organ. Convergence of CCAOs with the ECM of its metastatic organs provide a new platform for mechanistic study of cancer metastasis.

## Introduction

Despite achievements in early detection and treatment of primary tumors, 90% of current cancer-related death occur after the disease has metastasized. In bile duct cancer (cholangiocarcinoma, CCA), the 5-year survival rate is approximately 7-20% (1), and drops to 2% when the cancer metastasizes (2–4). Distant metastasis occurs relatively frequently in CCA over the course of a patient’s disease, ranging from 36.4-50.2% (5–7), with lung and lymph nodes the most common distant locations of metastasis (4, 6). Treatment options are surgical resection, liver transplantation, chemotherapeutics, intra-arterial treatments, and local ablative therapies, of which surgical intervention is the only potentially curative option (8). However, a large number of patients are not eligible, approximately 60-88%, including patients with distant metastatic loci (9). Understanding the micro-environmental cues of metastatic disease will aid in understanding the biology behind metastatic outgrowth and in developing novel therapeutic options for patients with unresectable CCA.

Tumor-derived organoids, consisting of primary epithelial cells grown as 3D structures, have emerged in recent years as highly promising biological disease models due to their self-renewal and self-organization capabilities, while maintaining the mutational landscape of the original tumor (10–12). CCA organoids (CCAOs) have been established as an attractive cellular source for various fundamental and translational biological applications, including identification of biomarkers, driver gene functionality testing, and drug screening (13–15). However, in a metastatic setting, micro-environmental cues at the target organ are crucial for cancer cell behavior, including colonization and proliferation of tumor cells (16, 17). These cues are not well recapitulated by current culture systems, as organoids are primarily cultured in basement membrane extracts (BME) derived from a mouse tumor (18). Particularly, the ‘seed and soil’ hypothesis, as posted by Paget in 1889, suggests that distant organs are different in their ability to provide a favorable environment (soil) for facilitating the growth of metastasized cancer cells (seeds) (19, 20). Progress in determining the role of the specific host organ, or ‘soil’, on the behavior of disseminated cancer cell, or ‘seeds’, aiming to colonize that organ is hindered by a lack of model systems that accurately recapitulate native organ structure.

As part of the micro-environment, the extracellular matrix (ECM) provides important biochemical and physical cues for tumor cell colonization (17). To isolate the ECM and study what the ‘soil’ comprises, decellularization methods can be applied. This technique uses enzymatic and/or chemical reagents to remove cells while preserving ECM characteristics, including architecture and protein composition (21–23). Decellularization has been established for many organs and tissues, including tumors. In previous work, we have shown that CCAO recellularization of decellularized liver and tumor scaffolds can unveil the influence of ECM on cancer-related processes, including growth, invasion, and chemo resistance. In CCA, location-specific metastases carry distinct prognostic values, with lymph node showing better prognostic outcomes compared to lung, however mechanistic insight into what is causing this is still unknown (4, 24). Therefore, modeling metastatic colonization in a tissue-mimicking structure that reflects *in vivo* micro-environmental cues is an outstanding challenge.

Here, we create an *in vitro* model encompassing patient-derived CCA organoids and decellularized human lung (dLu) and lymph node (dLN) to study metastatic cell-matrix interactions. The decellularized tissues were biochemically and biomechanically characterized, which revealed isolation of ECM components with an unique ECM protein signature for dLu and dLN and retention of tissue-specific function-related proteins. Recellularization of both decellularized tissues with CCAOs resulted in upregulation of different cancer stem cell populations, as determined by LGR5 and CD133, and an increased epithelial phenotype in dLN. Furthermore, CCAO grown in dLu and dLN had different proliferation patterns, influenced by both the original tumor and the ECM donor. These results illustrate the unique impact of the patient-derived tumor and the ECM of the target organ on key metastasis-related pathways and associated growth patterns.

## Materials and methods

### Sample procurement and tissue collection

CCA (n=3, including n=2 intrahepatic CCA and n=1 perihilar CCA) tissue samples were obtained from patients who underwent a curative-intent surgical resection, performed at the Erasmus MC in Rotterdam (**Table S1** for patient information). The Medical Ethical Council of the Erasmus MC approved the use of tissue for research purposes and patients provided written informed consent (MEC-2013-143). Samples were confirmed to be of tumor origin with histopathological assessment by a pathologist. CCA samples were stored at 4°C in Belzer UW cold storage solution (UW, Bridge to Life) and, if used for organoid initiation, processed within 24 hours after collection. Lung (n=3) tissue samples were obtained from donors for lung transplantation, performed at Sahlgrenska University hospital in Gothenburg, Sweden. Use of lung tissue was approved by the Swedish ethical review board in Lund (Dnr. 2008/413, 2011/581 and 2013/253). Lung tissue samples of peripheral lung was prepared by dissecting out cubes with a side length of approximately 10 mm with pleura remaining on one side and snap freezing them in isopentane chilled with liquid nitrogen. Lymph nodes (n=13) were obtained from donors who donated their liver for a liver transplantation procedure, performed at the Erasmus MC in Rotterdam (MEC-2014-060). The lymph nodes are hilar lymph nodes. Donor information for both lymph node and lung can be found in **table S2**. Lung and lymph node samples were initially stored at -80°C or -20°C and processed at a later stage for decellularization.

### Initiation and propagation of human patient-derived cholangiocarcinoma organoids

Initiation of CCAOs was done as previously described (13). Organoids were passaged in a 1:3-1:6 ratio approximately every 7 days, depending on their proliferation rate. Expansion medium (EM, **Table S3**) was refreshed every 3 or 4 days. Passaging was done by removing the EM and collecting the organoids in a 15 mL tube by adding ice-cold Advanced DMEM/F12 (AdvDMEM, Gibco) supplemented with 1% v/v penicillin-streptomycin, 1% v/v hepes, 1% v/v ultraglutamine, 0.2% v/v primocin) to the wells and scraping/pipetting. Subsequently, ± 8 ml ice-cold AdvDMEM was added to the 15 ml tubes. After centrifugation (453g, 5 min, 4°C), the supernatant was removed and the pellet was re-suspended in ice-cold AdvDMEM while mechanically breaking the organoids by pipetting up and down. After another centrifugation step (453g, 5 min, 4°C), the supernatant was removed and the cell pellet was re-suspended in BME (Cultrex). The mixture of cells and BME was plated in droplets of 25 µl in 12-, 24- or 48-well suspension culture plates (Greiner or Sarstedt) and cultured for 7 days before passaging occurred again.

### Decellularization procedure of human lymph nodes and lung tissue

Lung tissues were embedded in tissue-tek optical cutting temperature (OCT) compound, mounted on a metal holder and cut with a cryotome (Leica) at -15°C into 400 µm thick slices. 400 µm thick lung slices and entire lymph nodes were placed in a flask on a multi-position magnetic stirrer (**Figure S1A**). Lymph nodes were not cut into smaller slices before decellularization because of their relatively small surface area and disintegration of the lymph nodes during decellularization. Tissue samples were washed for 30 minutes with dH20, 1 hour with 9% hypertonic saline (NaCl) and again 30 minutes with dH20 to remove traces of blood, debris, and OCT compound by using osmotic effect. Thereafter, all tissue samples were decellularized with a solution consisting of 4% Triton-X-100 and 1% NH_3_ (hereafter referred to as TX-100 solution). TX-100 solution was replaced every hour for a total of 10 cycles including two overnight (O/N) cycles of approximately 16 hours, which resulted in transparent tissues. Subsequently, the tissues were washed with PBS (Gibco, HyClone) for 1 hour to remove traces of TX-100. Thereafter, tissues were incubated with DNase solution (2 mg/l DNase type 1 (Sigma) in 0.9% NaCl + 100mM CaCl_2_ + 100mM MgCl_2_) for 3.5 hours at 37°C on the magnetic stirrer. Finally, tissues were washed twice with PBS. Biopsy samples were taken before and after decellularization for various analysis. To note, two lymph nodes contained >50ng/mg wet tissue after decellulariation and were not included in subsequent experiments and/or analyses.

### Confirmation of decellularization procedure

Biopsies and decellularized tissue were fixed in 4% paraformaldehyde (PFA; Fresenius Kabi), solidified in 2% agarose in PBS, embedded in paraffin and sectioned at 4 µm using a microtome (HM 325). Slides of samples before and after decellularization were processed for routine histological stainings: hematoxylin and eosin (H&E), 4’,6-diamidino-2-phenylindole (DAPI; Vectashield, Vectorlabs), Masson’s Trichrome (TRI), Gomori’s (GOM), and picrosirius red (PSR; Sigma). Collagen type 1 is stained according to standard protocol by the pathology department (ErasmusMC, The Netherlands). Slides were imaged with a Zeiss Axioskop 20 microscope and captured with the Zeiss Axiocam 305 color or imaged and captured with Nanozoomer 2.0-HT (Hamamatsu). DAPI stained slides were analyzed using an EVOS microscope (Thermo Fisher Scientific). Additionally, DNA was isolated from lung and lymph nodes, before and after decellularization, using the QIAamp DNA Micro Kit (Qiagen) according to the manufacturer’s protocol. Subsequently, the total DNA content was measured using a Nanodrop spectrophotometer (Thermo Fisher Scientific; LU n=3, LN n=13) and corrected for the corresponding wet weight of the measured sample (ng DNA/mg wet tissue). The wet weight of the samples was determined before performing analysis.

### Collagen and sulfated glycosaminoglycan quantification

Total collagen content of lung and lymph nodes before and after decellularization (lung n=3 (T=0, T=decell); lymph node n=4 (T=0) and n=6 (T=decell)) was determined using a Total Collagen Kit (Quickzyme Biosciences) according to the manufacturer’s protocol. The absorbance of the collagen-binding dye was measured in a clear 96-well plate at 570 nm using an infinite M nano plate reader (Tecan). Background absorbance was subtracted. The content was corrected for the wet weight of the corresponding samples (µg collagen/mg wet weight tissue).

Sulfated glycosaminoglycan (sGAG) content of lung and lymph nodes before and after decellularization (lung N=3 (T=0, T=decell), lymph nodes n=4 (T=0) and n=6 (T=decell)) was determined using a Blyscan Sulfated Glycosaminoglycan Assay (Biocolor) according to the manufacturer’s protocol. Samples were digested in a Papain (Sigma) solution (10 mg/ml) at 65°C for 3 hours. The absorbance was measured in a clear 96-well plate at 656 nm using an infinite M nano plate reader (Tecan). The wet weight of the samples was weighted before performing analysis.

### Nanoindentation

The effective Young’s modulus (E) of decellularized tissue samples was measured using a Chiaro Nanoindenter (Optics11 Life) (**Figure S1B**). dLu (N=3) and dLN (N=3) were glued inside a 35 mm petri dish using NOA61 or NOA81 (Norland) and a UV torch (Walther Pro). The sample and probe were immersed in PBS before the measurement started. The stiffness of the probes used for dLu and dLN was respectively 0.027 N/m and 0.030 N/m. The probes had a tip radius of 3 µm and were ball shaped. First, the sample was indented to a depth of 2 µm in 4 seconds (0.5 µm/s). Then, the indentation was held at 2 µm for 1 second and finally the probe was retracted in 1 second. At least one matrix scan of 3x3 with a distance of 5 µm between indentation points was performed per decellularized extracellular matrix (dECM). The Hertzian contact model in the Optics 11 data viewer software (version V3.4.7) was used to calculate the effective Young’s Modulus (E) (25). Measurements with an unreliable model fit (R^2^<0.9) were considered as outliers and disregarded from further analysis.

### Rheology

A rotational rheometer (KINEXUS PRO; Technex) with a flat parallel plate geometry with a diameter of 20 mm was used to determine the Young’s modulus of the decellularized tissues (**Figure S1C**). All measurements were performed at 37°C and obtained by the rSpace software. dLu (n=3, 400 µm thick) and dLN (n=3) were placed on the bottom plate. Next, the top plate was lowered to a gap height of 2 mm (dLu1, dLu2), 3 mm (dLu3, dLN5) or 1.8 mm (dLN11, dLN10). First, the surface contact point was found by decreasing the gap at a rate of 0.01 mm/s with a measurement of the normal force every 0.01 second, which the software used to automatically determine the contact point. Subsequently, a shear oscillation frequency sweep (f: 10-0.01 Hz, slope: 10 points per decade) was performed to determine the viscoelastic properties of the samples. Subsequently, the dECM was compressed 4*10^−4^ mm every second for 13.3 minutes. Then, another shear oscillation frequency sweep (f: 10-0.01 Hz, slope: 10 points per decade) was performed. An approximation of the Young’s modulus (E) was determined over the whole range of compression. The strain was calculated by (h-h0/h0) where h is the gap while measuring and h0 is the initial gap when the surface contact point was found. The stress was calculated by (strain+1)*(Normal force/initial area) where the initial area was calculated by π*(sample radius)^2^, following the Cauchy stress calculations (26). In this way, the data was corrected for the size of each sample and increase in size after each compression step. The gradient slope over the whole range of compression resulted in an approximation of the Young’s modulus (E).

### Proteomic sample preparation

100 µL 50 mM Tris-HCl (pH 8.0) was added to the dECM scaffolds and the samples were snap frozen in liquid nitrogen, followed by homogenizing using a dismembrator. The sample was heated in a thermomixer for 5 min at 95°C. 90 µL 50 mM Tris-HCl and 5 µL 100 mM of 1,4-dithiothreitol were added and the sample was incubated at 50°C for 60 min. Subsequently, 5 µL 200 mM of 2-chloroacetamide was added and the sample was incubated at RT for 30 min. Then, 100 µL 50 mM Tris-HCl and 10 µL Peptide:N-glycosidase F (500 units/mL) was added and the samples were further incubated at 37°C for 4 h, followed by 5 min at 95°C. Finally, 25 µL sodium deoxycholate and trypsin was added (1:100, trypsin:protein) and the sample was incubated in a thermomixer O/N at 30°C and 1100 RPM. The next day, 25 µL 10% trifluoroacetic acid (TFA) was added to the sample, followed by 2X washes with ethylacetate: 300 µL ethylacetate (H_2_O saturated) was added, the mixture was mixed vigorously and then centrifuged for 2 min at 5,000 rpm. The upper layer was removed, followed by 45 min in the SpeedVac Vacuum Concentrator (Thermo Fisher Scientific) to evaporate the solvent and reduce the sample volume. The protein digest was desalted using C18 stage tips (Thermo Fisher Scientific). This was repeated for the flow through. The stage tip was then washed with 100 µL 0.1% TFA, centrifuged for 10 min at 2,000 rpm, followed by 2X elution of the peptides with 75 µL 50% acetonitrile (AcN) and centrifugation for 8 min at 2,000 rpm. Next, peptides were dried in the speedvac and reconstituted in 25 µL 2% AcN, 0.5% formic acid. Nanoflow liquid chromatography tandem mass spectrometry (nLC-MS/MS) was performed on an EASY-nLC coupled to an Orbitrap Fusion Lumos Tribrid mass spectrometer (Thermo), operating in positive mode. Peptides were separated on a ReproSil-C18 reversed-phase column (Dr Maisch; 15 cm × 50 μm) using a linear gradient of 0–80% acetonitrile (in 0.1% formic acid) during 90 min at a rate of 200 nl/min. The elution was directly sprayed into the electrospray ionization source of the mass spectrometer. Spectra were acquired in continuum mode; fragmentation of the peptides was performed in data-dependent mode by HCD.

### Proteomic data processing

Raw mass spectrometry data were analyzed using the Proteome Discoverer 2.3 software suite (ThermoFisher Scientific). The Mascot search algorithm (version 2.3.2, MatrixScience) was used for searching against the Uniprot database (taxonomy: Homo sapiens). The peptide tolerance was typically set to 10 ppm and the fragment ion tolerance was set to 0.8 Da. A maximum number of 2 missed cleavages by trypsin were allowed and carbamido-methylated cysteine and oxidized methionine were set as fixed and variable modifications, respectively.

### Proteomic data analysis

To identify and categorize the detected proteins that are related to the ECM, the dataset was compared to and filtered with the MatrisomeDB database (27). MatrisomeDB uses domain-based organization of matrisome-related proteins to obtain a complete collection of ECM proteomic data. Proteins identified are subdivided into ECM-affiliated proteins, secreted factors, collagens, ECM regulators, ECM glycoproteins, and proteoglycans. The identified matrisome proteins were further classified into 3 categories: a core ensemble of proteins, differentially expressed (DE) proteins, and exclusive proteins. The core ensemble of proteins consists of proteins that are present in all replicates of all conditions. Proteins are differentially expressed if the adjusted p-value is <0.05. Proteins are identified as ‘exclusive’ if they are present in samples of one condition, while absent in all replicates of another condition. To note, the difference between DE proteins and exclusive proteins is likely due to the sensitivity of mass spectrometry. Therefore, DE proteins and exclusive proteins were combined to apply enrichment analysis using the fgsea (version 1.16.0) R package.

### Preparation of decellularized matrices for organoid culture

One day before recellularization, scaffolds were washed with sterile PBS three times, followed by washing with AdvDMEM three times. Subsequently, the scaffolds were incubated overnight at 37°C with AdvDMEM, supplemented with 10x the concentration of penicillin- streptomycin, primocin and antiobiotic-antimycotic (Gibco) to avoid infections in the recellularization experiments. Decellularized matrices were washed three times with AdvDMEM on the day of recellularization. The scaffolds were placed in the middle of a new suspension or culture well plate (Greiner or Sarstedt) and were folded out as much as possible using a needle or tweezer.

### Recellularization

CCAOs (n=3) grown in BME droplets were harvested by removing the BME droplets from the wells using ice cold AdvDMEM as previously described. After removal of BME and AdvDMEM from the cell pellet, 1 ml trypsin (TrypLE, Thermo Fisher Scientific) was added. The suspension was incubated at 37°C for 15 minutes while applying mechanical disruption with a pipette every 5 minutes until organoid fragments were dissociated into small aggregates and single cells. Subsequently, 10 µl of the suspension was added to 10 µl Trypan Blue (Thermo Fisher Scientific) for cell counting using disposable cell counting chambers (Kova). After 15 minutes of incubation, trypsin was directly inactivated by the addition of cold AdvDMEM. The suspension was centrifuged for 5 minutes (453g, 4°C) and the resulting cell pellet was resuspended in EM to obtain the total amount of cells needed (approx. 200.000 cells/scaffold). Cells (5 µl) in EM were added to the dECM in a 12 or 24 suspension or culture well plate (Greiner or Sarstedt). The recellularized scaffolds were incubated for 3 hours at 37°C before adding 350-500 µl EM to the recellularized scaffolds. EM was refreshed every 3 or 4 days. Organoid cultures in BME were used as a control if appropriate.

### RNA isolation, cDNA synthesis and RT-qPCR

For RNA isolation, 3 or 4 scaffolds were added to 700 μl Qiazol. Qiazol lysed samples (3-4 recellularized scaffolds per replicate) were homogenized with a TissueRuptor (Qiagen). Messenger RNA was isolated with a miRNeasy kit (Qiagen) according to the manufacturer’s protocol. A Nanodrop spectrophotometer (Thermo Fisher Scientific) was used to measure RNA content. 500 ng complementary DNA (cDNA) was made by adding 5x PrimeScript RT Master Mix and dH2O to isolated RNA and inserted into a 2720 Thermal Cycler (Applied Biosystems) or SimpliAmp Thermal Cycler (Applied Biosystems). qPCR was performed according to standard procedures with 10 µl SYBR select master mix, 1 µl primers, 4 µl dH2O and 5 µl cDNA per reaction. All the tested primer sets are listed in (**Table S4**). The housekeeping genes Glyceraldehyde-3-Phosphate Dehydrogenase (GAPDH) and Hypoxanthine-guanine-phosphoribosyl-transferase (HPRT) were used as reference genes.

### Live/dead staining

CCAOs cultured in dECM were incubated in EM supplemented with 100 µg/ml Hoechst (Hoechst 33342, Thermo Fisher Scientific), 50 µg/ml propidium iodide (PI, Sigma-Aldrich) and 0.5 µM calcein (Calcein AM, Thermo Fisher Scientific) at 37°C for 30 minutes protected from light. Images were made with an EVOS FL fluorescent microscope (Thermo Fisher Scientific).

### Histological staining

PFA-fixed samples were solidified in 2% agarose in PBS. They were embedded in paraffin and sectioned at 4 µm using a microtome (HM325). Decellularized and recellularized scaffold slides were stained with H&E according to a standard protocol.

### Cell metabolic activity assessment

PrestoBlue™ Cell Viability Reagent (Thermo Fisher Scientific) was used to assess metabolic activity of CCAOs in dLu and dLN for the same sample at day 1, 4, 8, 11, 14 after recellularization. Presto Blue compound was diluted 10-fold (1:10) in EM, filtered and pre-warmed in a 37°C water bath for 10 minutes. EM was removed from the recellularized scaffolds and 500 µl Presto Blue solution was added to each well. The recellularized scaffolds were incubated at 37°C for 3.5 hours protected from light. Subsequently, the medium was plated in a 96 well plate in triplicate. The absorbance was measured using an CytoFluor Multi-Well plate Reader series 4000 (Perseptive Biosystems) with excitation of 530nm and emission of 590nm. Background absorbance was measured using non-recellularized dLu and dLN (both n=3). Background measurements were subtracted and data was normalized to day 1.

### Statistical analysis

Statistical analyses were performed using GraphPad Prism (version 9, GraphPad Software). Continuous unpaired variables between two groups were tested using a Mann-Whitney-U and presented graphically as means with standard deviation (SD). Kruskal-Wallis test was performed when more than two groups were compared. Two-way ANOVA test was performed for multi-variate analysis with multiple comparison testing for different time points or different donors (i.e. the sGAG/collagen contents). A description of the method and test results is noted if alternative statistical analysis was used. In all tests, a p value of <0.05 was considered significant.

## Results

### Decellularization of lymph node and lung tissue for isolation of a-cellular ECM scaffolds

**Figure 1:**
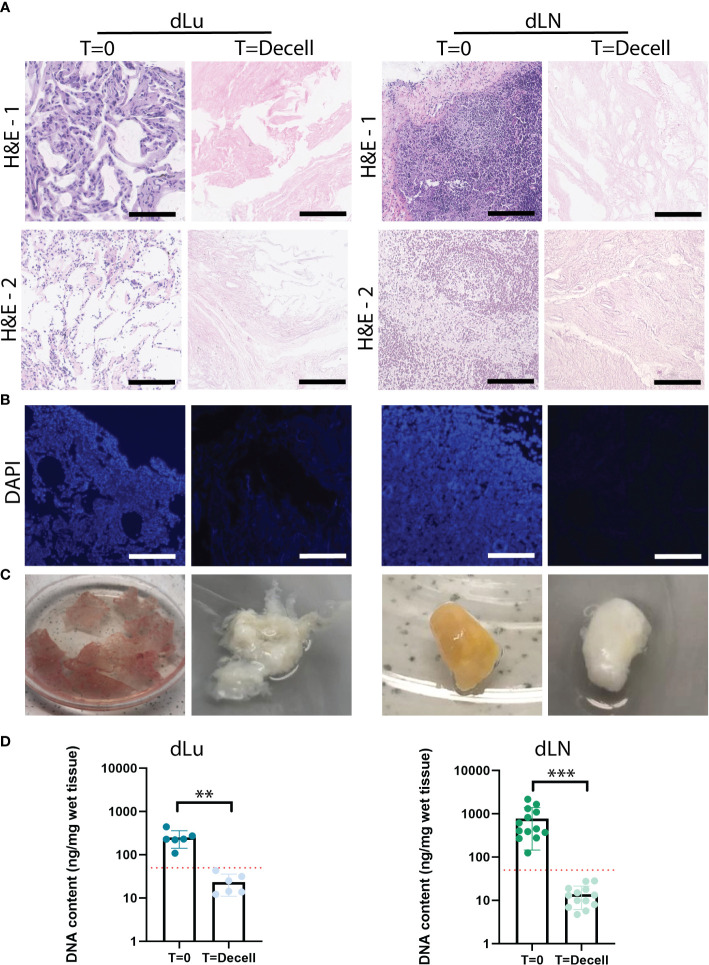
Extracellular matrix of lung and lymph nodes obtained by decellularization. **(A)** Representative H&E stainings of lung and lymph node before (T=0) and after (T=decell) decellularization show efficiently removal of cells from the scaffold and maintenance of ECM structure. Scale bars indicate 200 *µ*m. 1 and 2 show different donors for dLU (dLu2, dLu3) and dLN (dLN2, dLN12). **(B)** Representative DAPI stainings of lung (dLu2) and lymph node (dLN6) before and after decellularization confirmed removal of nuclear material from the scaffold. Scale bars indicate 250 *µ*m. **(C)** Lung slices of 400 *µ*m thick and an entire lymph node of 0.75 cm thick before and after decellularization, show the transformation in color from brown/yellow to translucent white. **(D)** Quantitative DNA content analysis of lung (n=3 patients, with each patient measured in technical duplicate, p=0.003) and lymph node (n=13, p=0.0002) before and after decellularization confirms successful decellularization. Error bars indicate ± SD. ** = p-value < 0.005, *** = p-value < 0.001. Paired t-tests were used for determining significance in DNA content. The red dotted line indicates a threshold of 50 ng DNA/mg wet tissue, which is a common criteria for adequate cell removal (29). For the DNA content dLu1-3 and dLN1-13 were used. .

To create tissue-specific *in vitro* metastatic colonization models for the lung and lymph node, we first decellularized lung (dLu) and hilar lymph nodes (dLN) (**Table S2**). An identical decellularization protocol was used for both tissue types. This is based on a previously described method for liver and liver tumor biopsy samples (28), so that recellularization would be minimally affected by the method of decellularization. Histological evaluation and DNA content quantification revealed successful decellularization (**Figure 1**). Lung sections show hollow structures of the alveoli and bronchiole before and after decellularization and preservation of ECM structure (**Figures 1A**, **S2**) (30). In lymph nodes, show the typical reticular meshwork architecture in T=0 conditions (**Figures 1A**, **S3**) (31). After decellularization, a relative dense structure lacking cellular material is observed, indicating preservation of overall tissue morphology. DAPI staining confirmed the removal of nuclear material from lung and lymph node (**Figure 1B**). Macroscopically, decellularization resulted in a white, translucent appearance for both lung and lymph node, as commonly seen for other decellularized organs as well (**Figure 1C**) (32, 33). Loss of nuclear material was confirmed by quantification of DNA. After decellularization, DNA content was significantly decreased in lung (p<0.0001) and lymph nodes (p=0.0022) (**Figure 1D**). Lymph node tissue has a higher cell density compared to lung, which is reflected by an average DNA concentration before decellularization in lymph node of 720ng DNA/mg wet tissue (n=13, SD: ± 569.8ng) and of 250.2ng DNA/mg wet tissue (n=6, consisting of 3 patients and 2 technical replicates, SD: ± 98.7ng) in the lung. After decellularization, the DNA content is reduced to 19.9ng DNA/mg wet tissue (n=13, SD: ± 17.6ng) for dLN and 23.3ng DNA/mg wet tissue (n=6, SD: ± 11.2ng) for dLu, equaling a reduction of 97.2% and 90.7%, respectively. Both absolute values and percentage reduction (i.e. <50 ng DNA/mg wet tissue and 90% reduction in DNA content) adhere to common criteria for complete cell removal (29). Thus, utilizing the same method, both lung and lymph node tissue was successfully decellularized.

### Decellularized scaffolds show retention of ECM-related components

**Figure 2:**
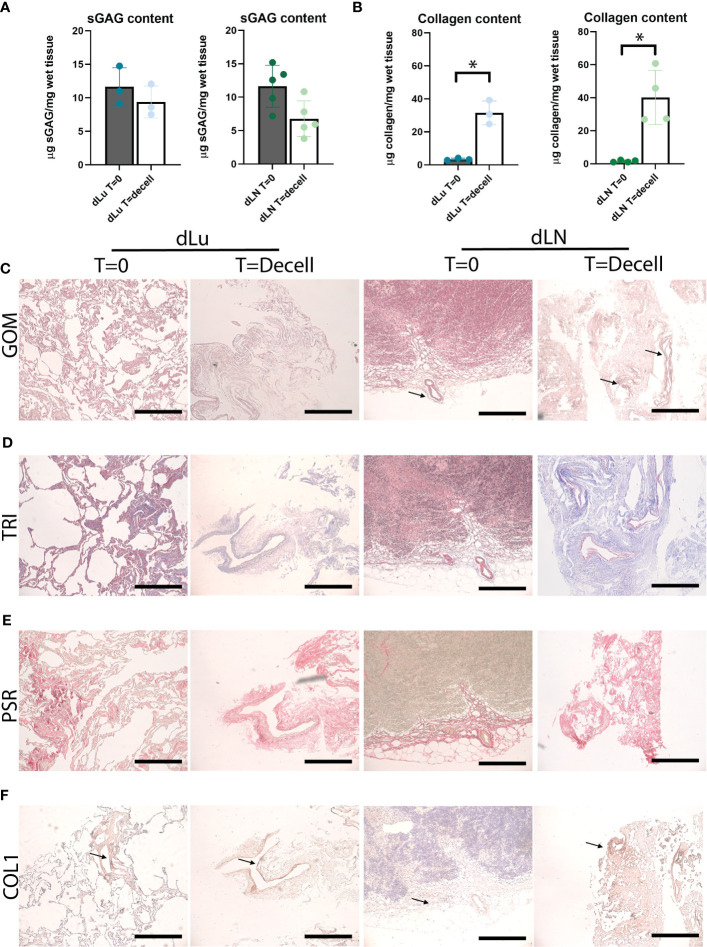
Preservation of ECM proteins after decellularization of human lung and lymph node. **(A)** Quantitative sGAG content analysis of lung (n=3, p=0.5) and lymph node (n=5, p=0.07) before and after decellularization showing retention of sGAG. dLu1, 2, 3 and dLN17, 27, 7, 10, 11 were used. **(B)** Quantitative collagen content analysis of lung (n=3, p=0.02) and lymph node (n=4, p=0.02) before and after decellularization, showing retention of collagen. dLu1, 2, 3 and dLN2, 4, 7, 15 were used. Paired t-tests were used for determining significance for determining significance in sGAG and collagen content. *= p-value <0.05. **(C–E)** Lung (dLu1) and lymph node (dLN12) before (T=0) and after (T=decell) decellularization stained with Gomori’s (GOM, **C**), Masson’s Trichrome (TRI, **D**) and PicroSirius Red (PSR, **E**) shows ECM architecture primarily consisting of collagen fibers. GOM shows reticulin (black), nuclei (red), and cytoplasm (pink). TRI shows muscle (red), collagen (blue), nuclei (brown/black), cytoplasm (brick red). **(F)** Representative images of collagen type 1 staining of lung (dLu1) and lymph node (dLN1) before and after decellularization. All scale bars indicate 200 µm.

To further characterize the decellularized scaffolds, the level of retention of ECM-related components was assessed. Sulfated glycosaminoglycans (sGAG) are important regulators of various cancer-related processes, including angiogenesis, invasion, proliferation and metastasis (34, 35). For both lung and lymph nodes, sGAGs were preserved after decellularization. The total sGAG content for both lung and lymph node per mg wet tissue decreased slightly, with a 1.4-fold and 1.9-fold decrease for dLu and dLN, respectively (**Figure 2A**). The slight difference between the tissues could be related to the higher cell density in dLN, which means relatively more cell-associated sGAGs are lost during the process of decellularization. Subsequently, collagen content was assessed, as collagen is the primary structural component of the ECM. The collagen concentration increased for both lung (before decellularization: 3.36 µg/mg wet tissue, SD± 0.54; after decellularization: 31.49 µg/mg wet tissue, SD± 5.94) and lymph node (before decellularization: 1.53 µg/mg wet tissue, SD± 0.58; after decellularization: 43.46 µg/mg wet tissue, SD± 15.63) (**Figure 2B**). Gomori’s staining shows the presence of reticular fibers in lymph node tissue at T=0 and after decellularization (**Figure 2C**). The relatively high retention of collagen was confirmed by histological staining’s (PicroSirius Red and Masson’s Trichrome), with abundant positivity in both dLu and dLN (**Figures 2D, E**). In the lung, collagen type 1 is important for mechanics and confers primarily tensile properties, while in the lymph nodes it is part of the reticular fibers (30, 31). **Figure 2F** demonstrates diffuse abundance and presence of collagen type 1 after decellularization (black arrows). Overall, decellularization of human lung and lymph nodes resulted in preserved components of the ECM and in acellular scaffolds.

### Divergent mechanical properties of decellularized lung and lymph node scaffolds

**Figure 3:**
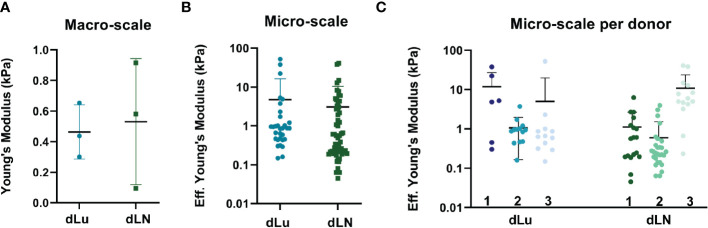
Macroscopic and microscopic mechanical characterization of dLu and dLN. **(A)** Macroscopic compression measurements showing the Young’s Modulus of dLu (n=3, dLu1, dLu2, dLu3) and dLN (n=3, dLN11, dLN10, dLN5). **(B)** Effective Young’s Modulus measured by micro-indentation (3x3 matrix scans, 5µm between indentations with a total measured area of 15x15 µm) of dLu (n=3 donors) and dLN (n=3 donors). **(C)** Effective Young’s Modulus measured by micro-indentation split per donor for both dLu (dLu1, dLu2, and dLu3) and dLN (dLN11, dLN10, dLN5). Each data point is a different region of the sample obtained in a 3x3 matrix scan.

Collagens forms a three-dimensional network, and its architecture is central to tissue functioning through providing mechanical properties (36). Therefore, to determine both macro- and microscopic mechanical properties of dLu and dLN, rheology measurements and micro-indentation were performed, respectively. Macroscopic properties of dLu and dLN were determined by assessing rheological properties under compression. The approximation of the Young’s Modulus (E), determined over the whole range of compression, for was 0.46 ± .18kPa for dLu and 0.53 ± .41kPa for dLN (**Figure 3A**). More heterogeneity in macroscopic stiffness is seen in dLN. On a micro-scale, by obtaining the effective Young’s modulus by micro-indentation, the stiffness values ranged from 0.15-52.3 kPa for dLu and 0.05-40.9 kPa for dLN (**Figure 3B**). The effective Young’s modulus is defined as the Young’s modulus without making any assumptions regarding Poisson’s ratio. This heterogeneity is also captured on a per donor basis, but did not show any significant differences between donors for dLu or dLN (**Figure 3C**). Thus, on a micro-scale a similar trends persists, with a large standard deviation indicating heterogeneity in the stiffness for both tissue types, and heterogeneity in macroscopic stiffness for dLN in particular.

### Decellularized tissue scaffolds contain common and divergent ECM proteins

**Figure 4:**
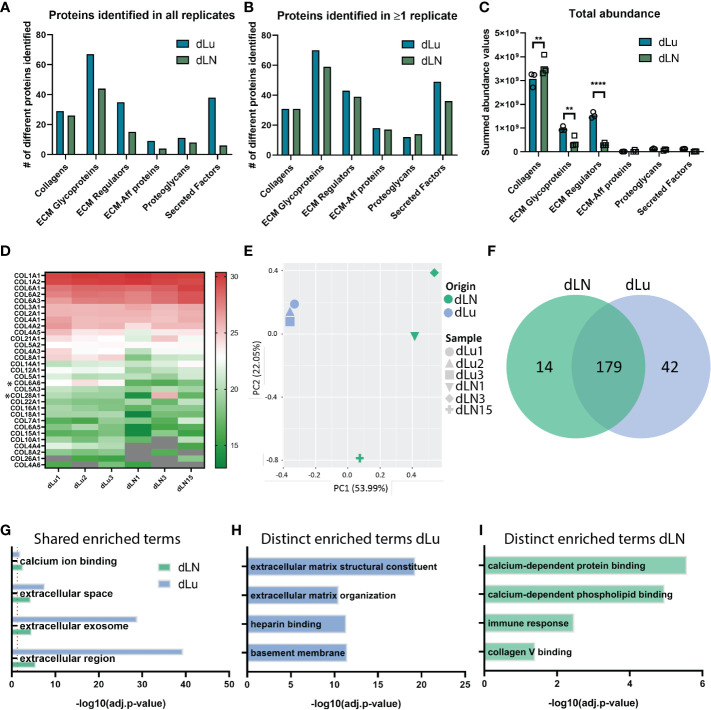
Analysis of global ECM proteome derived from decellularized lung and lymph node tissue. **(A, B)** Global matrisome protein composition identified by Mass Spectrometry displaying the total number of unique proteins identified in all biological replicates **(A)** or identified in at least 1 biological replicate **(B)**. Only proteins overlapping with MatrisomeDB classification for ECM-related proteins are included. **(C)** Total abundance values per ECM-related protein category. A two-way ANOVA with multiple comparisons per matrisome category statistical analysis was performed (Collagen p=0.006; ECM Glycoproteins p=004, ECM Regulators p<0.0001). **(D)** Heat map displaying the relative log_2_(abundance) values for all identified collagen subunits. Grey color indicates that no abundance value was present. *p < 0.05. **(E)** Scatter plot based on principal component analysis (PCA) displays a global separation between ECM protein composition of dLN and dLu. **(F)** Exclusive and shared proteins identified in dLu and dLN. **(G–I)** Enrichment analysis of selected biological processes and pathways for protein abundance differences as displayed in **(E)** for dLu and dLN. Shared enriched processes are processes that are significantly enriched in both decellularized tissues, distinct enriched processes are processes that are exclusively enriched in either dLu or dLN. For all mass spectrometry analysis dLu1, dLu2, and dLu3 were used for lung, and dLN1, dLN3, and dLN13 were used for lymph node. **= p-value < 0.005; **** = p-value < 0.0001.

We next examined whether the heterogeneity in mechanical properties was also represented in the overall protein composition. For this, mass spectrometry was employed to find shared and divergent ECM proteins in dLu and dLN. ECM-related proteins were categorized following the matrisome classification standards: collagen, glycoproteins, ECM regulators, ECM-affiliated proteins, proteoglycans, and secreted factors (27). Analysis of the proteome identified proteins in all categories, highlighting the complexity of the dECM in both tissues (**Figures 4A, B**). The most abundant proteins in both dLN and dLU are largely overlapping, particularly collagens are present in both decellularized scaffolds (**Figure S4**). However, important differences in highly abundant proteins are also present, including elastin (ELN) in the lung, which is important for lung development and alveolar formation (37). Still, most differences are present in lower abundant ECM-related proteins, highlighting the complexity of the environments that are provided by decellularized scaffolds (**Suppl. File 2**). Filtering for proteins present in all biological replicates showed that dLu contained a larger variety of proteins in all categories compared to dLN (**Figure 4A**). This is similar when filtering for ECM proteins only present in one replicate, although the difference between dLu and dLN becomes less apparent, indicating a higher level of intra-dLN heterogeneity in protein composition (**Figure 4B**). The larger diversity in ECM glycoproteins and ECM regulators in dLu is translated to a significantly higher total abundance, based on summed tryptic peptide intensities (**Figure 4C**). However, dLN had a significantly higher total collagen abundance compared to dLu, congruent with the collagen quantification based on acid hydrolysis (**Figures 4C**, **2B**). Surprisingly, dissecting the higher abundance per collagen subtype and subunit does not reveal major differences, with only COL6A6 (p=0.02) and COL28A1 (p=0.03) significantly upregulated in dLu compared to dLN (**Figure 4D**). Principal component analysis revealed tissue-specific signatures of ECM proteins by segregation of dLu and dLN clusters based on PC1 (**Figure 4E**). Furthermore, dLN showed a higher overall heterogeneity across donors, primarily displayed by PC2, which is in line with the higher heterogeneity in stiffness across donors (**Figures 4E**, **3A**). The different signatures are also represented by exclusive expression of 42 proteins in dLu and 14 proteins in dLN (**Figure 4F**). Next, these uniquely identified proteins were used for enrichment analysis with the DAVID bioinformatics tool (38). The enriched terms were divided into shared (**Figure 4G**) and distinct (**Figures 4H, I**). As expected, extracellular space (GO:0005615) and region (GO:0005576), similar enrichment terms both containing extracellular proteins, are enriched in both dLu and dLN, as ECM is isolated through decellularization (**Figure 4G**). The distinct enriched terms reveal retention of tissue-specific functions in the decellularized scaffolds, with enrichment of immune response in dLN, as it function is primary immune system-related, and basement membrane enrichment in dLu, which in native lung is crucial for functioning of gas exchange through binding endothelium and epithelium together (**Figures 4H, I**) (39). Altogether, dLu and dLN have distinct protein signatures, with high protein diversity and reflect tissue-specific functional aspects.

### CCAOs grown in dLu and dLN scaffolds attain tissue-specific expression of cancer-related genes

**Figure 5:**
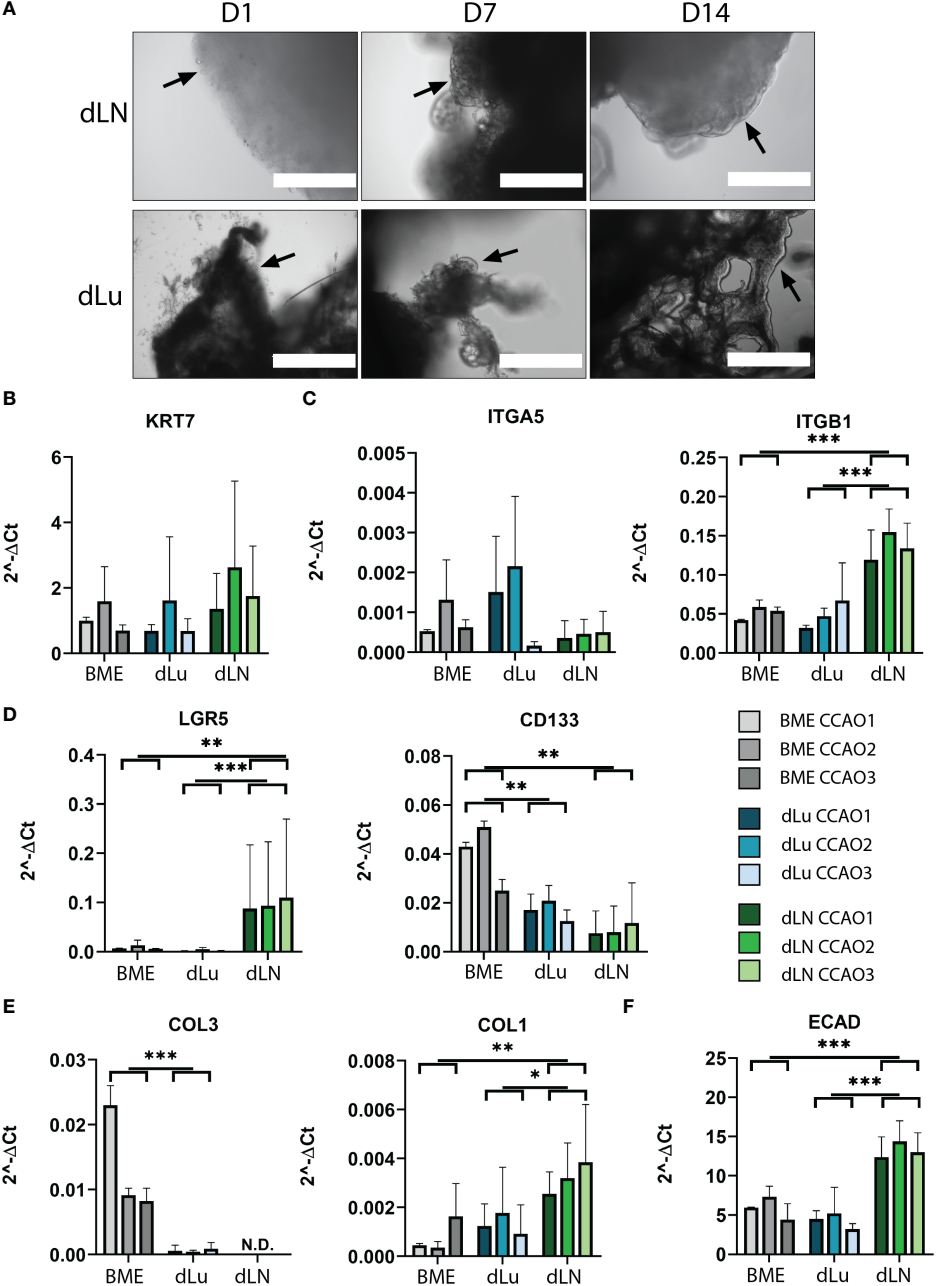
Recellularization of dLu and dLN with CCAOs reveals distinct gene expression profiles. **(A)** Representative bright field microscopy images of CCAOs cultured in dLu and dLN on day 1, 7 and 14 after recellularization. Day 1 scale bar indicates 1000 *µ*m, day 7 and day 14 scale bars indicate 400 *µ*m. Black arrows indicate the progression from single cells at day 1 to a complete cellular layer at day 14. **(B)** Gene expression of KRT7 in CCAOs for BME control and recellularized dLu and dLN. **(C)** Gene expression of ITGA5 and ITGB1 in CCAOs for BME control and recellularized dLu and dLN. **(D)** Gene expression of LGR5 and CD133 in CCAOs for BME control and recellularized dLu and dLN. **(E)** Gene expression of COL3A1 and COL1A1 in CCAOs for BME control and recellularized dLu and dLN. **(F)** Gene expression of ECAD in CCAOs for BME control and recellularized dLu and dLN. * = p-value < 0.05, ** = p-value < 0.005, *** = p-value < 0.0001. N.D. means that the values were not detectable. Mann-Whitney U statistical test was used for determining significance in gene expression profiles. All gene expression profiles were normalized to GAPDH. For recellularization experiments dLu1, dLu2, dLu3 were used for lung and dLN9, dLN8, and dLN1 were used for lymph node.

Next, we assessed the effect of the decellularized ECM scaffolds on CCAOs, to mimic metastatic outgrowth in lung and lymph node. Patient-derived CCAOs (n=3) were cultured in BME, harvested, and seeded on dLu and dLN. CCAOs grown in BME were spherical in shape and proliferated over time as previously described (**Figure S5**) (40). In dLu and dLN, bright field microscopy images showed the adhesion of single cell and clumps of CCAOs to the ECM observed at day 1 of recellularization. Subsequently, organoid-like structures appeared around day 7, which transformed into a complete cellular layer surrounding the edge of the ECM scaffolds at day 14 (**Figure 5A**).

To identify the biological processes that are important for metastasis of CCA in the lung and lymph nodes, gene expression profiles of CCAOs cultured in dLu, dLN, and BME were compared. As expected, KRT7, a marker of cholangiocyte-lineage (41), was comparable between all conditions and showed high expression, indicating retention of CCA phenotype (**Figure 5B**). Integrin β1 (ITGB1) and integrin α5 (ITGA5), both ECM binding subunits of integrin receptors (42–44), were probed for their expression profiles (**Figure 5C**). ITGB1 was significantly upregulated in dLN (versus both dLu and BME p=0.03) revealing tissue-specific cell-ECM interactions. ITGA5 showed high heterogeneity in expression between different CCAOs, with a 13-fold increase of CCAO2 vs CCAO3 in dLN (p=0.1). This suggests that in lymph node metastasis upregulation could be patient-dependent. LGR5 and CD133, both markers of (different) cancer-stem cell subpopulations (45, 46), were significantly affected by the ECM (**Figure 5D**). LGR5 was significantly upregulated in dLN compared to dLu (p<0.001) and BME (p=0.005), while CD133 was significantly higher in BME compared to both decellularized scaffolds (both p=0.016). Thus, this indicates that there is a tissue-specific involvement of cancer-stem cell populations in metastatic outgrowth in CCA. Furthermore, significant higher expression of COL1A1 in dLN (vs BME p=0.0075, vs dLU p=0.013) and COL3A1 in BME (vs dLU p=0.007) indicates that the reciprocal production of ECM proteins by tumor cells is also affected by the ECM of the metastatic organ (**Figure 5E**). Additionally, epithelial-to-mesenchymal transition (EMT), and the reverse process of mesenchymal-to-epithelial transition (MET) are thought to play a role in metastatic dissemination and subsequent colonization, respectively (47, 48). ECAD was significantly upregulated in dLN (vs dLu p<0.001, vs BME p=0.002), indicating (re)expression induced by the extracellular microenvironment, possibly due to the tumor cells undergoing MET (**Figure 5F**). Classical EMT-markers VIM and SNAI1 showed heterogeneous expression (**Figures S6A, B**). The ECM also influences gene expression profiles of matrix modulating genes (**Figures S6C**, **D**). Tissue inhibitor of metalloproteinases 1 and 2 (TIMP1, TIMP2) are significantly upregulated in dLN compared to dLU (TIMP1 p=0.026, TIMP2 p=0.04 only for CCAO2), while metalloproteinases 2 and 9 (MMP2, MMP9) show varied expression profiles in the different decellularized scaffolds. Overall, various cancer-related processes, including cancer stem cell plasticity, ECM production, cell-ECM binding, and EMT/MET, are influenced by the extracellular environment of the target metastatic organ in a tissue-specific pattern.

### Metastatic outgrowth of CCAOs is ECM and patient dependent

**Figure 6:**
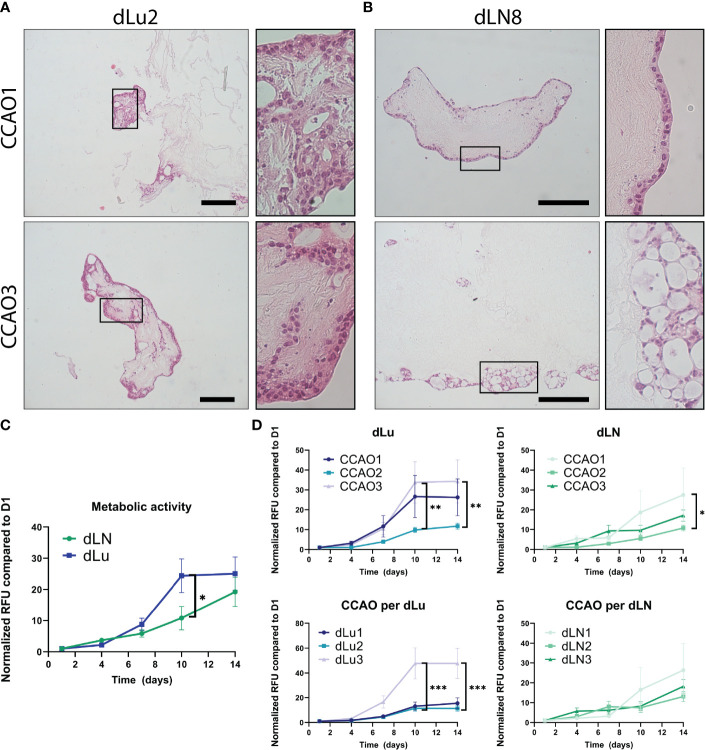
Proliferation and migration dynamics of metastasis in CCAOs. **(A, B)** Representative H&E stainings of CCAO1 (top) and CCAO3 (bottom) in dLu **(A)** and dLN **(B)**. The black rectangle shows a higher magnification image of the morphology of the CCAOs in the decellularized scaffolds. Scale bars indicate 250 *µ*m. **(C)** Metabolic activity measurements of recellularized dLN (n=27, n=3 ECM donors with n=3 CCAO lines and n=3 technical replicates per combination) and dLu (n=27, n=3 ECM donors with n=3 CCAO lines and n=3 technical replicates per combination) consecutively measured on day 1, 4, 7, 10, and 14. All Relative Fluorescent Units (RFU) data is normalized to day 1. **(D)** Metabolic activity measurements for CCAOs in dLU split based on patient origin of CCAO (i.e. separated CCAO1, CCAO2, and CCAO3) and donor of decellularized scaffolds (i.e. separated dLu1, dLu2, and dLu3). **(E)** Metabolic activity measurements for CCAOs in dLU split based on patient origin of CCAO (i.e. separated CCAO1, CCAO2, and CCAO3) and donor of decellularized scaffolds (i.e. separated dLN1, dLN2, and dLN3). * = p-value<0.05, ** = p-value<0.005, *** = p-value<0.0001. Multiple t-test were used together with Holm-Sidak correction method to correct for multiple comparisons. For recellularization experiments dLu1, dLu2, dLu3 were used for lung and dLN9, dLN8, and dLN1 were used for lymph node.

In a metastatic setting, after reaching the microenvironment of the distant organ, cancer cells will colonize the niche and often display a state of dormancy before changing to a state of proliferation and outgrowth (49, 50). We therefore examined if the ECM plays a role in the change from dormancy to outgrowth, and the effect on the associated cell migration and proliferation dynamics. H&E staining of CCAOs cultured in dLN and dLu showed cell-ECM attachment, with the occurrence of different invasive patterns (**Figures 6A, B**). In dLu, CCAOs exhibited localized colonization, with extensive in-growth in the scaffold at these locations, reminiscent of the localized growth pattern *in vivo* (**Figure 6A**) (51). CCAOs in dLN colonized primarily the outer rim, either in single-cell or cellular clumps, with an epithelial-like phenotype and limited scaffold in-growth (**Figure 6B**). This is congruent with the upregulation of ECAD in CCAOs cultured in dLN compared to dLu (**Figure 5F**) and the upregulation of TIMPs in dLN compared to dLU suggesting that the inhibition of matrix degradation is associated with the limited invasion occurring (**Figure S6C**). In all, decellularized scaffolds of lung and lymph node can induce different migratory patterns.

Subsequently, a metabolic assay was used to probe the metabolic activity of the tumor organoids over time in each condition. As a control, BME-cultured CCAOs exhibited significant increase in metabolic activity over 14 days, as expected and reported in literature (**Figure S7A**, (13)). CCAOs in dLN (n=27, n=3 ECM donors with n=3 CCAO lines and n=3 technical replicates per combination) and dLu (n=27, n=3 ECM donors with n=3 CCAO lines and n=3 technical replicates per combination) also showed an increase in metabolic activity over time (**Figure 6C**). However, a different growth pattern was observed in both metastatic locations, with a significant delay in metabolic activity increase after 10 days in dLN compared to dLu (10.8x increase in dLU vs 24.4x increase in dLN, p=0.014). After 14 days, no significant difference was observed (p=0.97), indicating that it was a delay in growth, rather than a consistently lower growth rate. Dissecting the role of seed (i.e. the CCAO) and soil (i.e. the ECM) reveals that in lung metastasis, both seed and soil have a significant influence on metastatic outgrowth (**Figure 6D**). This is exhibited by CCAO3 and Lu3 showing significantly higher metabolic activity after 10 and 14 days when comparing tumor and donor scaffold, respectively. For lymph node metastasis, this effect was less evident, with CCAO3 having a significantly larger increase in metabolic activity after 7 days, suggesting an earlier switch from dormancy to outgrowth in this case (**Figure 6E**). No ECM-dependent differences were found in dLN (**Figure 6E**). To note, no difference in initial seeding efficiency was observed between dLN and dLu, as represented by absolute metabolic activity values at day 1 (**Figure S7B**). In summary, these data suggest that the dynamics of outgrowth after colonization are multi-factorial, both patient and ECM related. In this model, metaphorically both the “seed” and “soil” influence metastatic growth of cancer cells in the lung, while in the lymph node the growth is dictated primarily by the seed (cancer cell).

## Discussion

The process of cancer metastasis consists of a multi-step cascade during which tumor cells disseminate from the primary tumor, survive in the lymphatic or blood circulation, and colonize distant organs. The tumor cells are heavily influenced by the various microenvironments that they encounter during this cascade, including, but not limited to, the ECM of the target organ for metastasis (52–54). Particularly, the interaction of seed (i.e. cancer cells) and soil (i.e. ECM) that is encountered in the metastatic organ plays a role in the dynamics of metastatic colonization (20, 55). Herein, we show the possibility to obtain a tissue-specific metastatic model by converging decellularized human lung and lymph nodes with patient-derived CCAOs to investigate the role of the ECM in metastatic outgrowth. We demonstrated the capability to decellularize human derived tissue of distant metastatic locations for CCA and reveal the biomechanical and biochemical characteristics of dLu and dLN, which recapitulate the tissue of origin. Furthermore, dLu and dLN scaffolds support adhesion and culture of CCAOs while stimulating distinct, tissue-specific gene expression profiles. The associated growth patterns further delineate the role of both seed and soil in the outgrowth of colonized metastatic CCA, with dLu inducing a significantly higher proliferation rate compared to dLN.

The decellularization method employed in this study, utilizing Triton X-100, was able to successfully eliminate cellular material from both human lung and lymph node tissue. The resulting decellularized scaffolds recapitulated the composition of native ECM, with enrichment of basement membrane-related proteins in dLu and immune system-related proteins in dLN. Decellularization of human lymph nodes has not been reported yet in literature, but employing an identical decellularization method as lung provided comparative scaffolds for studying cell-ECM interactions in these respective organs.

The mechanical role of ECM in cancer metastasis is highly diverse, affecting matrix remodeling, cell spreading, migration and metastasis (53, 56, 57). Therefore, biomechanical characterizations of the decellularized tissue were obtained, which indicated similar stiffness for dLu and dLN, with a notable variability in macro-scale stiffness for dLN. Although the obtained mechanical properties (Young’s Modulus) of dLu are comparable to literature, these properties are only known for animal-derived decellularized ECM, non-decellularized human ECM or engineered hydrogels (58–62), extending the relevance of this study. Mechanical characterization of human lymph node ECM is absent in literature. The heterogeneity in mechanical properties in dLN is mimicked by a diversity in ECM proteins, showing the correlation between mechanical and chemical properties of the extracellular environment. To note, causation is not inferred, as not only molecular composition, but also cross-linking, spatial heterogeneity, and alignment of ECM architecture can contribute to the heterogeneity observed in mechanical properties.

Cell adhesion to ECM is crucial for the process of metastasis, and integrins are the main cell adhesion receptors that facilitate these functions. In multiple cancer types, integrin β1 signaling plays a crucial role in metastatic colonization and outgrowth (63). In dLN, CCAOs upregulate integrin β1, indicating that the role of integrin β1 in CCA metastasis is organ-dependent. Furthermore, the production of ECM-proteins, and their associated proteases, by tumor cells in a metastatic environment can remodel the environment (64, 65). COL1A1 is upregulated in a lymph node environment, which coincides with findings in breast cancer, where collagen 1 fiber density was increased in lymph node metastasis, and lung cancer, where COL1A1 expression highly correlated with lymph node metastasis (66, 67). E-cadherin, an epithelial marker, is also upregulated in dLN. This is corroborated by the epithelial-phenotype present in dLN, as well as the observed limited invasion. In other tumors, an epithelial phenotype is often associated with formation of secondary tumors, with E-cadherin-positive metastatic foci (68, 69). The associated lack of invasion in dLN could be due to the absence of cellular interactions normally present in the lymph node during the process of metastasis, including interactions between resident immune cells and recruited bone marrow-derived cells (70, 71). Incorporation of these cell types in this system would allow for even deeper understanding of metastatic colonization by modelling the interactions between primary tumor, immune cells, and secondary target sites.

Combining multiple decellularized scaffold donors with patient-specific organoids allows for delineating the role of both seed and soil in cancer metastasis. Importantly, after arrival at a distant metastatic organ, cancer cells will colonize the niche and often initiate a dormant phenotype (49). Dormancy licenses the cancer cells to survive this novel environment through chemotherapeutic resistance (i.e. less cellular division means less susceptibility to conventional chemotherapy) and immune cell avoidance, mediated by downregulation of MHC-1 expression (72). Here we show that the ECM can dictate the timing and duration of this dormancy phase, whereby dLN (2.9-fold increase from day 4 to 10) resulted in a slower increase in metabolic activity compared to dLu (11-fold increase from day 4 to 10). The cause of the switch from dormancy to proliferation is complex, and this study shows that the ECM in isolation can influence this process.

Lymph node colonization might not be a final destination for metastasis, and could contribute to further distant metastases including lung. The frequency of cells metastasizing from the lymph node to different distant organs is dependent on the cancer-type, and still a topic of debate (73, 74). Mechanistically, cancer cells are able to colonize lymph nodes, invade lymph node blood vessels and subsequently colonize the lung (75). In our study, CCAOs cultured in dLN exhibited a significant upregulation of LGR5, a well-recognized stem cell marker (76, 77), compared to both dLu and BME. LGR5 marks tumor-initiating cells with a cancer stem cell-phenotype in liver cancer (77) and these cancer stem cells are thought to be responsible for tumor progression, including metastasis (78). Thus, the high level of LGR5 in CCAOs that colonize the lymph node suggests that there is a pool of cancer stem cell-like cells present which could be responsible for migration from the lymph node to the lungs. This is congruent with the clinical observation that lymph node metastasis often precedes lung metastasis in CCA patients (24), and the association of LGR5 expression with lymph node metastases in other tumor types (76, 79).

For the lung, donor-dependent proliferation differences were observed, with dLu3 favoring proliferation compared to dLu1 and dLu2. To note, this lung was obtained from a current smoker, which is in contrast with the other two donors (former smoker <10 years and never smoker). Although the relationship between smoking and metastasis of CCA has not been studied, it is known that smoking affects the initiation and progression of multiple other cancers such as soft tissue sarcoma, esophageal cancer, breast cancer colorectal cancer, and lung cancer (80–82). Further research is necessary to establish a direct relationship between smoking and metastasis of CCA to the lungs, given the dependence on multiple variables.

In summary, acellular scaffolds of human lung and lymph nodes were successfully obtained *via* decellularization. Biochemical and biomechanical characterization revealed the retention of tissue-specific characteristics, as well as expanded our understanding of the mechanical properties of the ECM. Subsequent recellularization revealed differences in CCA metastatic colonization in the lung and lymph nodes through gene expression profiles and proliferation dynamics. Converging organoids with organ-specific decellularized ECM provides a valuable tool for probing cell-matrix interactions in a metastatic setting.

## Data availability statement

The datasets presented in this study can be found in the PRIDE database (https://www.ebi.ac.uk/pride/ - accession number: PXD038191) or in the **Supplementary Material**.

## Ethics statement

The studies involving human participants were reviewed and approved by Medical Ethical Council of the Erasmus MC and the Swedish ethical review board in Lund. The patients/participants provided their written informed consent to participate in this study.

## Author contributions

GT, LL, MV designed the study. MV, LL and GK obtained funding. HR, IS, GW-T, OR aided in the collection of tissues. IS collected patient information. GT, MB performed experiments. GT, JT, JD aided in the conception and implementation of mass spectrometry experiments, and JT and GT performed the analysis. GT, MB, JC, IM, GK set-up mechanical characterization of decellularized scaffolds, with MB and IM conducting rheological measurements and MB and JC conducting nanoindentation measurements. GT, MB, IS, JC performed data analysis of mechanical measurements. GT, MB collected all data and drafted the figures. GT, MB, LL, MV wrote the manuscript. All authors critically reviewed and revised the manuscript.
