# Supplementary material for: Modelling metastatic colonization of cholangiocarcinoma organoids in decellularized lung and lymph nodes
Source: Front Oncol. 2023 Jan 18;12:1101901. doi: 10.3389/fonc.2022.1101901 (PMC9890173; doi:10.3389/fonc.2022.1101901)
Supplement: Supplementary file 1 [file DataSheet_1.docx]

Supplementary data to manuscript:

“Modelling metastatic colonization of cholangiocarcinoma organoids in decellularized lung and lymph nodes” by Gilles S van Tienderen^1^, Marije van Beek^1^, Ivo Schurink^1^, Oskar Rosmark^2^, Henk Roest^1^, Jantine Tieleman^3^, Jeroen Demmers^3^, Iain Muntz^4^, Gunilla Westergren-Thorsson^2^, Gijsje Koenderink^4^, Luc JW van der Laan^1^, and Monique MA Verstegen^1^*


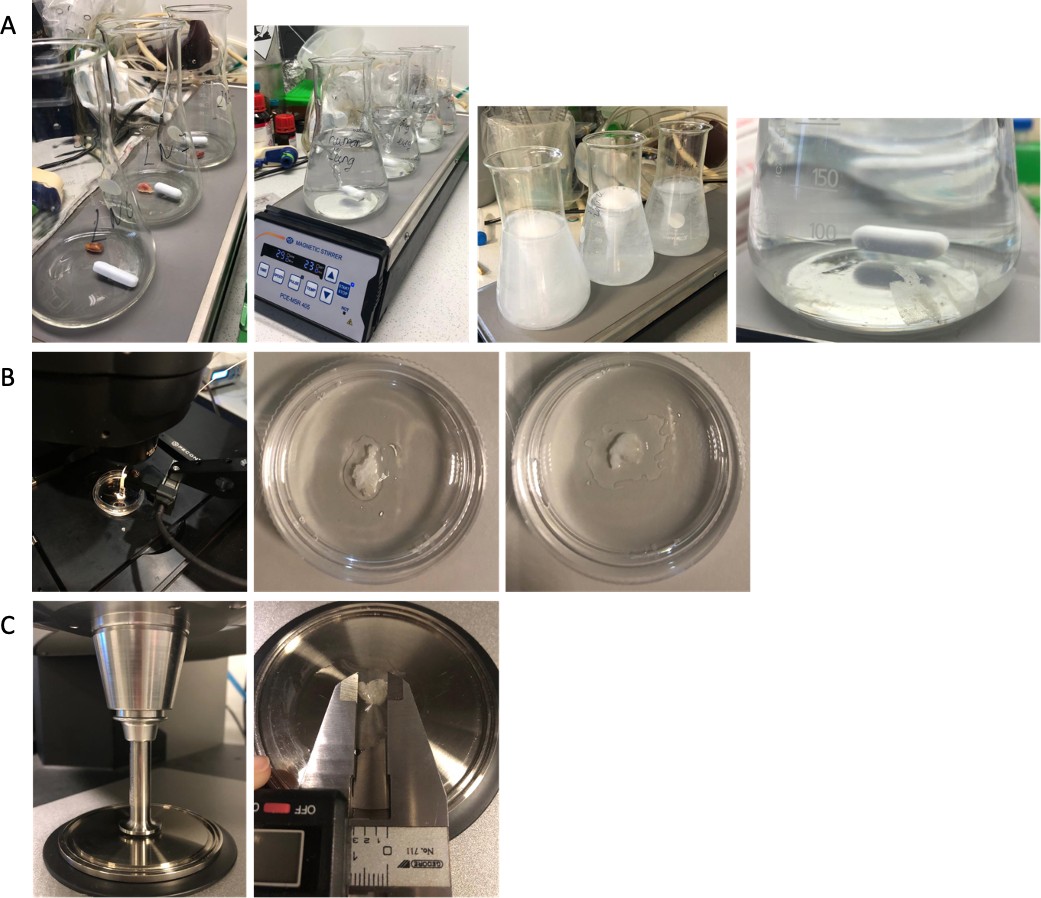


**Figure S1: Decellularization process and experimental set-up for mechanical characterization of decellularized tissues**. **A)** Decellularization process for lung tissue and lymph nodes in an Erlenmeyer flask on a multi-position magnetic stirrer. **B)** Nanoindentation set-up mounted on an inverted optical microscope (left) and glued samples inside a 35 mm petri dish (right). **C)** Rheology set-up with two parallel stainless steel plates (left) and sample width measurement (right).

**Figure S2: Histological evaluation of human lung tissues at T=0 (before decellularization).** Scale bar represents 200 μm. GOM = Gomori’s, TRI = Massons’s Trichrome, PSR = PicroSirius Red (collagen, red), and Collagen Type I (COL 1).


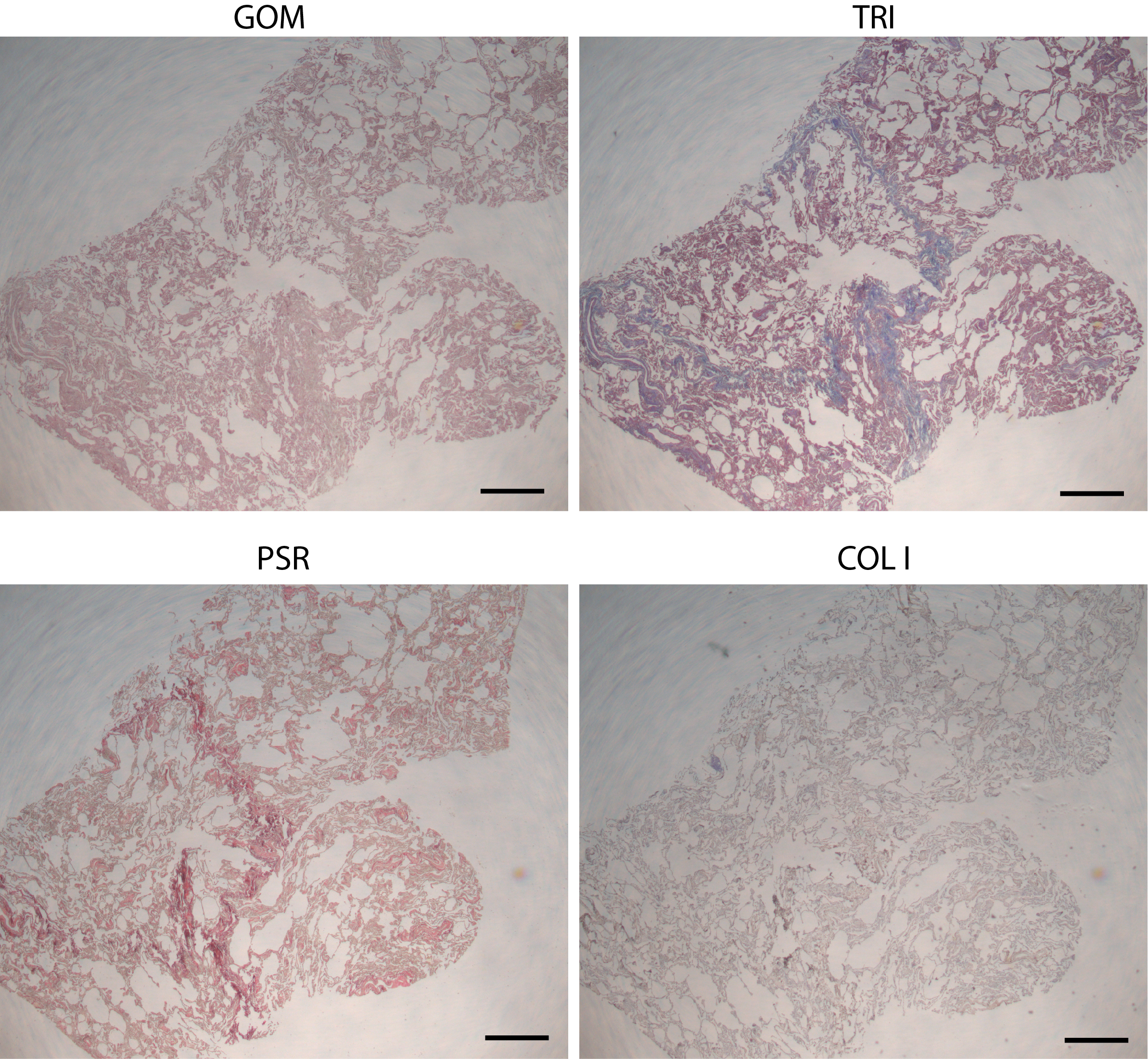

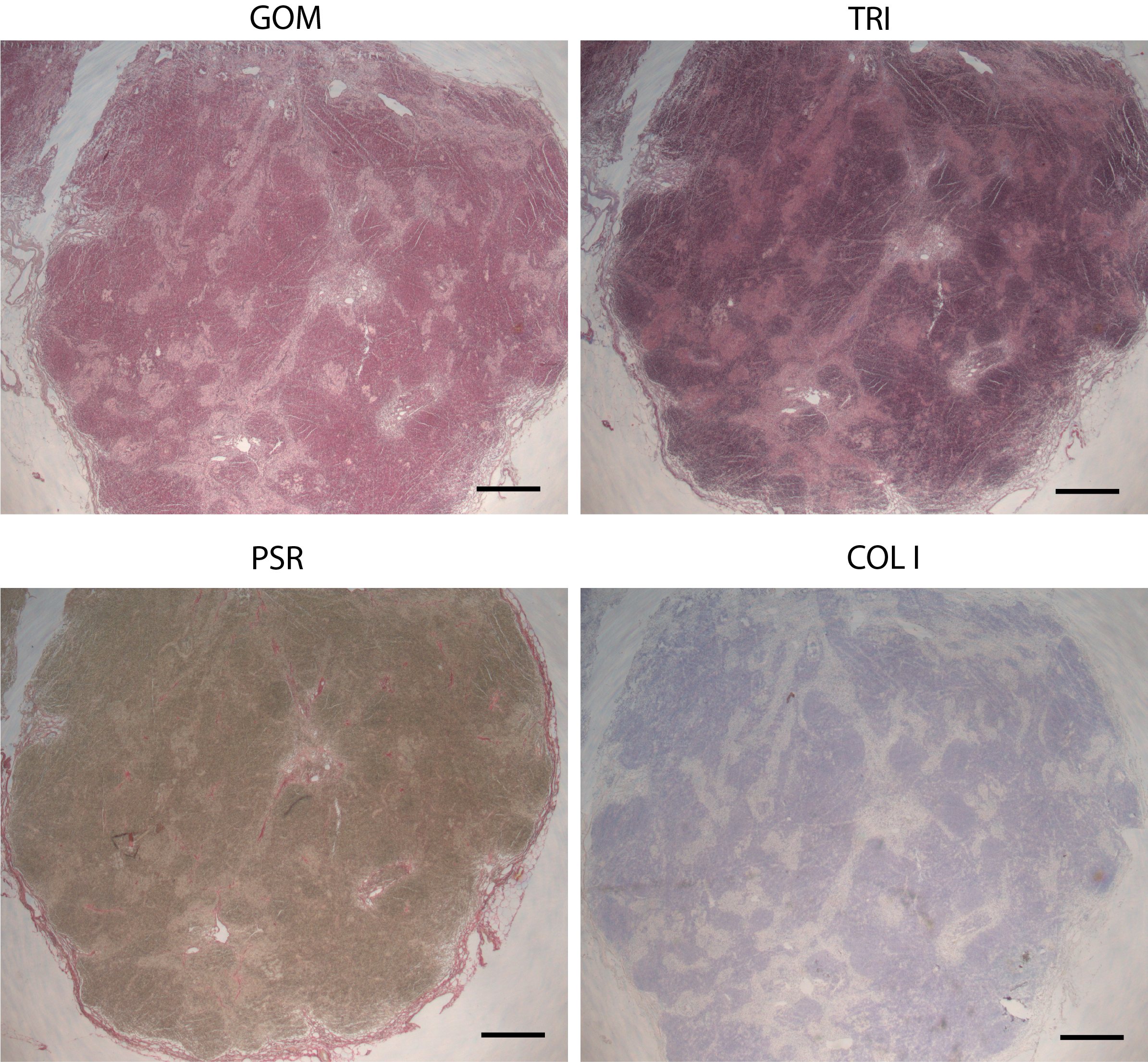


**Figure S3: Histological evaluation of human lymph node tissues at T=0 (before decellularization).** Scale bar represents 200 μm. GOM = Gomori’s, TRI = Massons’s Trichrome, PSR = PicroSirius Red (collagen, red), and Collagen Type I (COL 1).


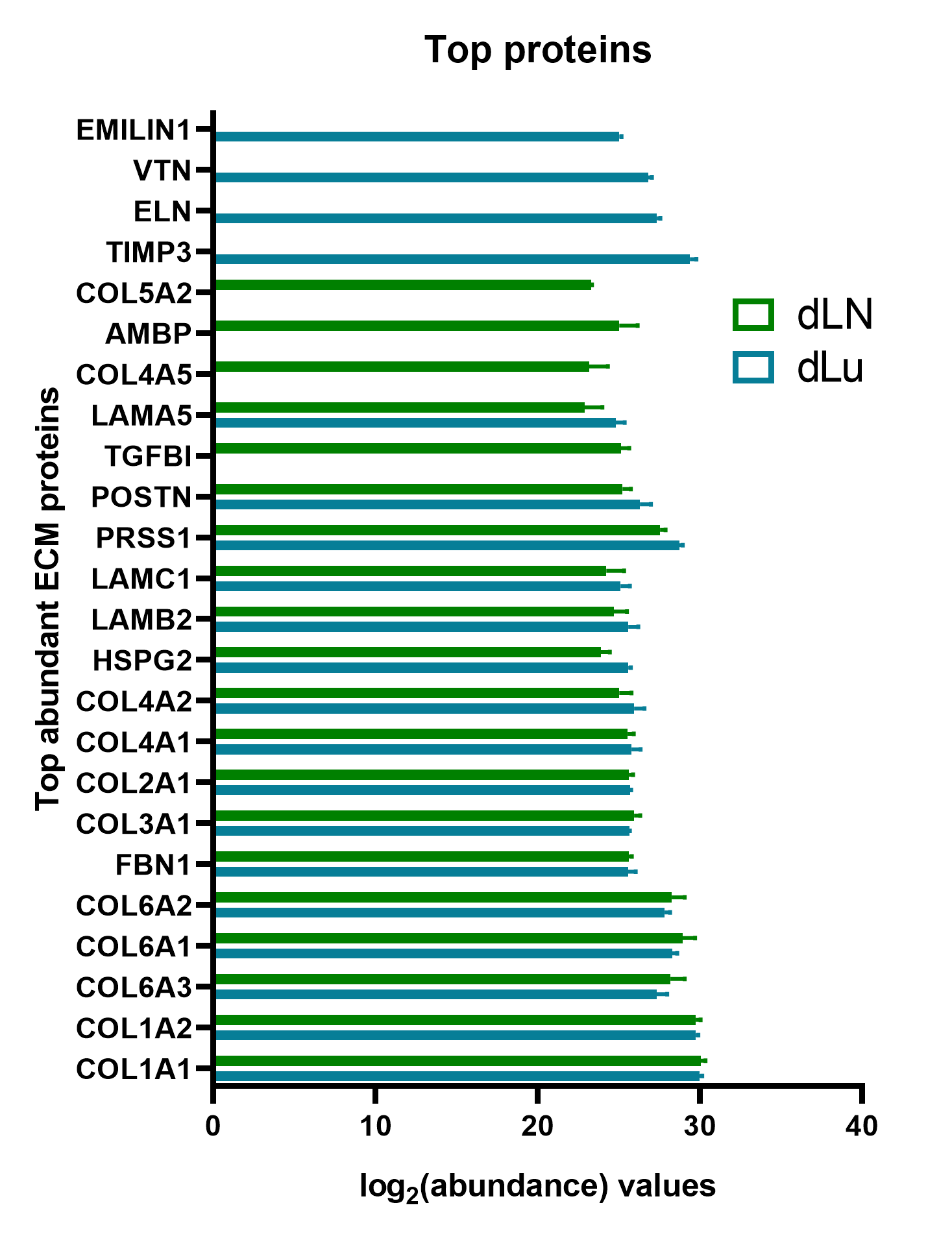


**Figure S4: Analysis of the top 20 most abundant ECM-related proteins.** Average log_2_(abundance) values for decellularized lung and lymph nodes reveal the most abundant proteins. These consist primarily of collagens that are present in both decellularized tissues. However, both dLu and dLN contain different ECM-related proteins as well within the top 20 most expressed proteins.

**Figure S5: Expansion of CCAOs.** Bright field microscopy images of CCAOs (n=3), growth

from day 0 after splitting until day 7 in BME. Scale bars indicate 2 mm. Shown is one representative experiment while passaging.


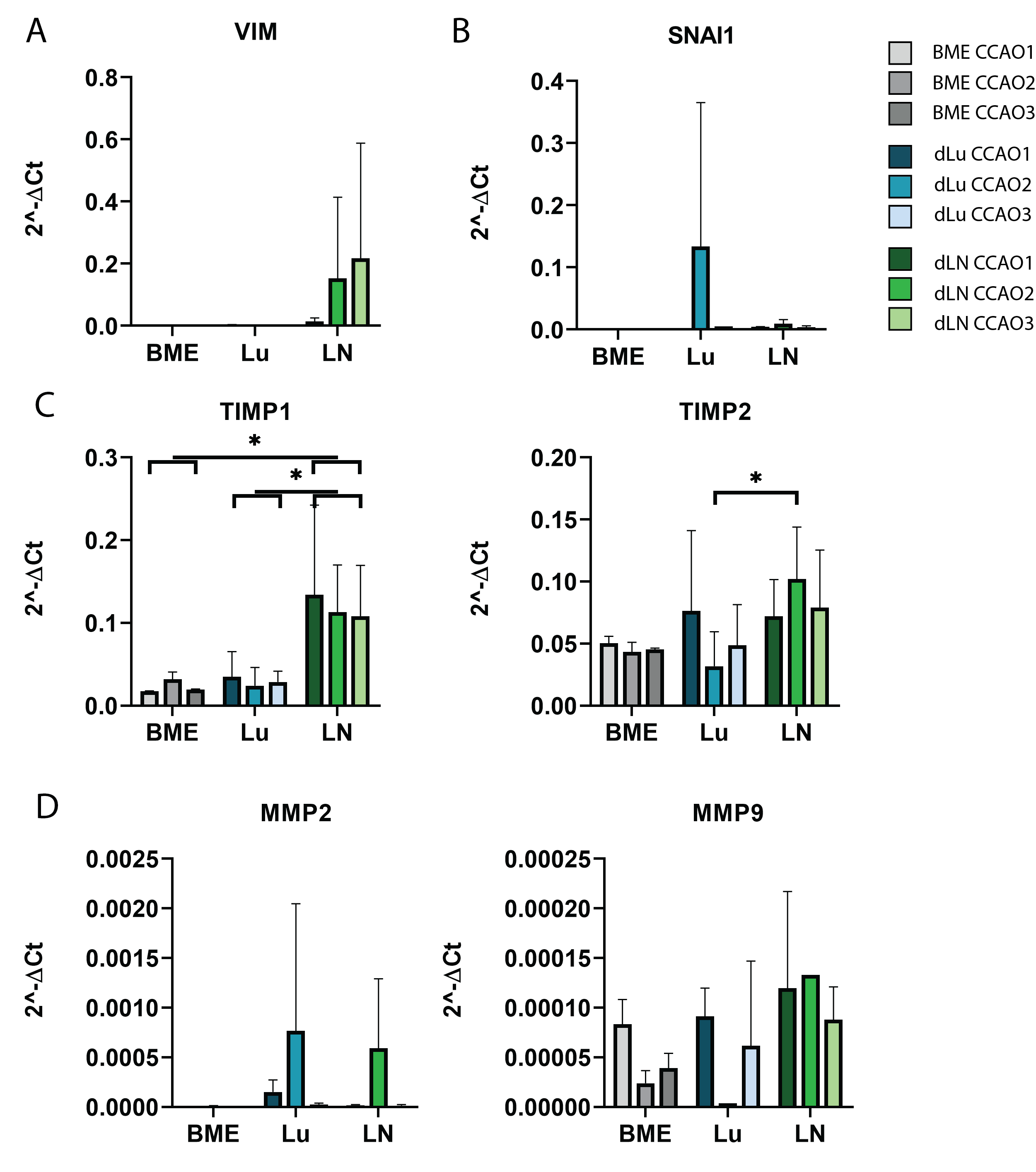


**Figure S6: Gene expression profiles of CCAOs cultured in BME, dLu, and dLN for A)** Vimentin, **B)** SNAI1, **C)** TIMP1 and TIMP2, and **D)** MMP2 and MMP9. Shown is the mean plus SD of the three individual CCAO lines. Differences were not statistically significant.


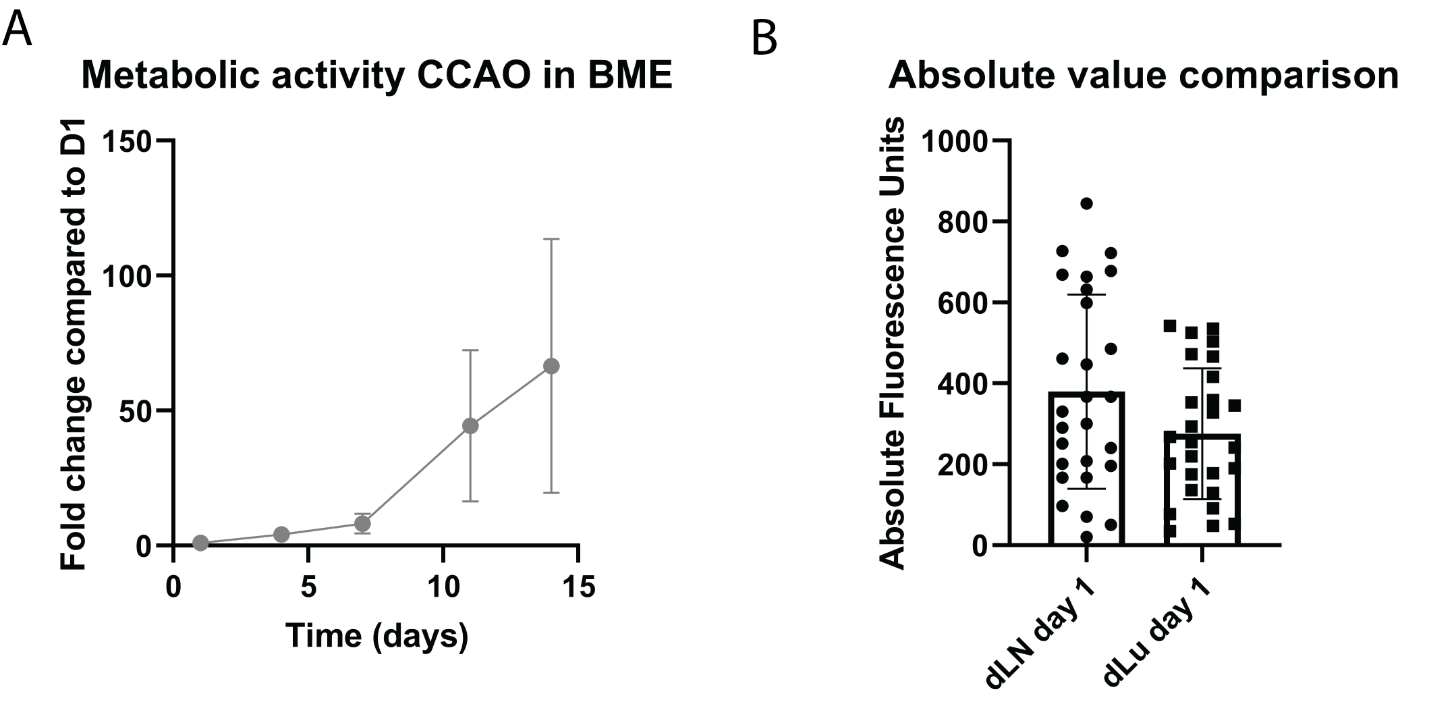


**Figure S7: Supplemental data on metabolic activity of CCAO. A)** Metabolic activity measurements of BME-cultured CCAO (n=3) consecutively measured on day 1, 4, 7, 10, and 14. All RFU data is normalized to day 1. Shown is mean with error bars indicating SD. **B)** Absolute value comparison of CCAO metabolic activity at day 1 for dLN and dLu, revealing no difference (p=0.14). Error bars indicate SD.

**Supplemental Table S1: Patient information and characteristics for the CCAOs.**

| **Sample** | **Gender** | **Age** | **Localisation** | **Histological grade** |
| --- | --- | --- | --- | --- |
| CCAO1 | F | 34 | Perihilar | Moderate-well differentiated |
| CCAO2 | M | 60 | intrahepatic (segment 4) | Moderate-well differentiated |
| CCAO3 | F | 77 | intrahepatic (segment 4) | Poor-moderate differentiated |

**Supplemental Table S2: Donor information and characteristics for lymph node and lung.**

| **Lymph node/Lung** | **Donor^1^** | **Sex (M/F)** | **Age** | **BMI** | **Smoking** | **Pack years^2^** |
| --- | --- | --- | --- | --- | --- | --- |
| **LN01** | DBD | F | 58 | 21 | No | 0 |
| **LN02** | DCD | F | 55 | 26 | No | 0 |
| **LN03** | DBD | F | 44 | 25 | Yes | 12 |
| **LN04** | DBD | M | 82 | 27 | No | 0 |
| **LN05** | DBD | M | 47 | 24 | No | 0 |
| **LN06** | DBD | M | 55 | 23 | Yes | 5 |
| **LN07** | DBD | M | 50 | 26 | Yes | 15 |
| **LN08** | DBD | F | 31 | 26 | No | 0 |
| **LN09** | DCD | M | 51 | 24 | No | 0 |
| **LN10** | DBD | F | 57 | 28 | No | 0 |
| **LN11** | DBD | M | 58 | 21 | No | 0 |
| **LN12** | DBD | F | 55 | 15 | Yes | 50 |
| **LN13** | DBD | F | 63 | 21 | Yes | 13 |
| **Lu01*** | DBD | M | 66 | NA | Yes | >10 |
| **Lu02** | DBD | M | 68 | NA | No | 0 |
| **Lu03** | DBD | M | 66 | NA | Yes | NA |

**^1^Human donor type: Donation after Cardiac death (DCD), Donation after Brain death (DBD).
^2^Pack year is defined as 20 cigarettes smoked every day for one year.
*former smoker.
NA: Not Available.**

**Supplemental Table S3: Medium components for Expansion Medium (EM)**

| **Component** | **Concentration** | **Brand** |
| --- | --- | --- |
| Adv+ |  | Gibco |
| N2 | 1% | Gibco |
| B27 | 2% | Gibco |
| N-Acetylcystein | 1mM | Sigma-Aldrich |
| gastrin | 10 nM | Sigma-Aldrich |
| EGF | 50 ng/ml | Peprotech |
| FGF10 | 100 ng/ml | Peprotech |
| HGF | 25 ng/ml | Peprotech |
| nicotinamide | 10nM | Sigma-Aldrich |
| A83.01 | 5 µM | Tocris |
| Forskolin | 10 µM | Tocris |
| R-Spondin | 10% | Conditioned medium |

**Supplemental Table S4: Primers used in this study.**

| **Genes** | **Forward primer sequence 5’ to 3’** | **Reverse primer sequence 5’ to 3’** |
| --- | --- | --- |
| **CD133** | CCTGGGGCTGCTGTTTATTA | ATCACCAACAGGGAGATTGC |
| **COL1A1** | CCCAGGTCCCCCTGGAAAGA | CCCGGCAGCACCAGTAGC |
| **COL3A1** | TACCAAGGACCCCCTGGTGAA | GGTCGTCCGGGTCTACCTGAT |
| **ECAD** | CTGGACAGGGAGGATTTTGA | ACCTGAGGCTTTGGATTCCT |
| **FN1** | CTTTTGCGTCGCCAGCCGAG | GCATGAAGCACTCAATTGGGCA |
| **GAPDH** | ACCAGTCAACAGGGGACATAA | CCAGGCGCCCAATACGACCA |
| **HRPT** | AGGGTCGGGGGCTTCAACTTA | CTTCGTGGGGTCCTTTTCACC |
| **ITGA5** | GGACGCCGCGCGGAAAAGAT | CACCCACAATTTGGCCCTGCT |
| **ITGB1** | CTACGGATTATACCTGGCCTTCC | AGGAAGCTGGATACGGATGTCA |
| **LGR5** | CGGGAGAAATTGCAGGAGG | TGCTGTTCCTGAATCTGAGC |
| **KRT7** | AGAGAAGTTCAGTGCCCAGC | TGACATCCTGTCCCTGAGTG |
| **VIM** | CGGGAGAAATTGCAGGAGG | TGCTGTTCCTGAATCTGAGC |
| **SNAI1** | GCGAGCTGCAGGACTCTAAT | GCCTCCAAGGAAGAGACTGA |
| **TIMP1** | CCCAGAGAGACACCAGAGAACC | GTCCCCACGAACTTGGCCC |
| **TIMP2** | CAGTGTGTGGGGTCTCGCTG | AGCGCGTGATCTTGCACTCG |
| **MMP2** | CAACTACGATGATGACCGCAA | GTGTAAATGGGTGCCATCAGG |
| **MMP9** | CTCTGGAGGTTCGACGTG | GTCCTGGCAGAAATAGGCTT |
